# A Comparison of Health Professionals’ and Patients’ Views of the Importance of Outcomes of Bariatric Surgery

**DOI:** 10.1007/s11695-016-2186-0

**Published:** 2016-05-02

**Authors:** Karen D. Coulman, Noah Howes, James Hopkins, Katie Whale, Katy Chalmers, Sara Brookes, Alex Nicholson, Jelena Savovic, Yasmin Ferguson, Amanda Owen-Smith, Jane Blazeby, On behalf of the By-Band-Sleeve Trial Management Group, Jane Blazeby, Richard Welbourn, James Byrne, Jenny Donovan, Barnaby C. Reeves, Sarah Wordsworth, Robert Andrews, Janice L. Thompson, Graziella Mazza, Chris A. Rogers

**Affiliations:** 1 School of Social and Community Medicine, University of Bristol, Canynge Hall, 39 Whatley Road, Bristol, BS8 2PS UK; 2 Department of Upper GI and Bariatric Surgery, Taunton and Somerset NHS Foundation Trust, Taunton, Somerset TA1 5DA UK; 3 The National Institute For Health Research Collaboration for Leadership in Applied Health Research and Care West (NIHR CLAHRC West), University Hospitals Bristol NHS Foundation Trust, Whitefriars, Lewins Mead, Bristol, BS1 2NT UK; 4 Division of Surgery, Head & Neck, University Hospitals Bristol NHS Foundation Trust, Upper Maudlin Street, Bristol, BS2 8HW UK

**Keywords:** Outcomes, Patient views, Health professional views, Quality of life, Survey, Core outcome sets

## Abstract

**Background:**

A comprehensive evaluation of bariatric surgery is required to inform decision-making. This will include measures of benefit and risk. It is possible that stakeholders involved with surgery value these outcomes differently, although this has not previously been explored. This study aimed to investigate and compare how professionals and patients prioritise outcomes of bariatric surgery.

**Methods:**

Systematic reviews and qualitative interviews created an exhaustive list of outcomes. This informed the development of a 130-item questionnaire, structured in four sections (complications of surgery; clinical effectiveness; signs, symptoms, and other measures; quality of life). Health professionals and patients rated the importance of each item on a 1–9 scale. Items rated 8–9 by at least 70 % of the participants were considered prioritised. Items prioritised in each section were compared between professionals and patients and interrater agreement assessed using kappa statistics (*ĸ*).

**Results:**

One hundred sixty-eight out of four hundred fifty-nine professionals (36.6 %) and 90/465 patients (19.4 %) completed the questionnaire. Professionals and patients prioritised 18 and 25 items, respectively, with 10 overlapping items and 23 discordant items (*ĸ* 0.363). Examples of items prioritised by both included ‘diabetes’ and ‘leakage from bowel joins’. Examples of discordant items included ‘re-admission rates’ (professionals only) and ‘excess skin’ (patients only). Poor agreement was seen in the ‘quality of life’ section (0 overlapping items, 8 discordant, *ĸ* −0.036).

**Conclusions:**

Although there was some overlap of outcomes prioritised by professionals and patients, there were important differences. We recommend that the views of all relevant health professionals and patients are considered when deciding on outcomes to evaluate bariatric surgery.

**Electronic supplementary material:**

The online version of this article (doi:10.1007/s11695-016-2186-0) contains supplementary material, which is available to authorized users.

## Introduction

Surgery for severe and complex obesity (bariatric surgery) can lead to substantial improvements in patients’ health. It may be evaluated using many different outcomes, including clinical endpoints such as improvements in diabetes and cardiovascular risk, and patient-reported outcomes (PROs) such as quality of life. Information on outcomes of bariatric surgery is important to inform future patient and clinician decision-making. Previous systematic reviews have documented the large number of outcomes of bariatric surgery reported in studies, with 1088 clinical outcomes and 68 different validated patient-reported outcome (PRO) measures identified [1, 2]. This creates problems for synthesising data across studies in meta-analyses. This heterogeneity of published outcomes may be partially explained by the different views and values held by different stakeholders involved in the care of patients with severe obesity.

Studies in other clinical areas, including multiple sclerosis, rheumatology, oncology, and breast reconstruction surgery, have examined differences in how stakeholders value outcomes [3–9]. Patients in these studies often placed more priority on quality of life outcomes than clinicians, particularly outcomes related to emotional and psychological functioning [3–9]. As patients are the users of healthcare services, it is recommended that their views are included in the evaluation of healthcare interventions such as surgery, and this may be particularly relevant following elective procedures such as bariatric surgery [10–12]. Understanding how professionals and patients value outcomes of bariatric surgery has not been previously investigated. The aim of this study was to explore and compare how health professionals and patients prioritise outcomes of bariatric surgery.

## Methods

The work reported here was integrated into the development of a core outcome set for bariatric surgery [13–15]. A core outcome set is a minimum set of outcomes to be reported in all trials of a particular disease/condition [14]. Use of a core outcome set can help to improve the consistency of outcomes reported across trials and facilitate meta-analyses [14]. The development of the core outcome set consists of three stages. Stage 1 established an exhaustive list of outcomes which was used to inform the development of a questionnaire survey. Stage 2 used the questionnaire to survey key stakeholders’ views on the importance of the outcomes. Stage 2 also includes a second and third survey incorporating Delphi methods to provide participants with anonymous feedback of others’ views (from the previous survey round) to inform subsequent prioritisation of the outcomes. Stage 3 involves face-to-face meetings of stakeholders to reach consensus about the most important outcomes to be part of a core outcome set. The results of the first survey were used to investigate and compare health professionals’ and patients’ views of important outcomes (this study). As such, only stage 1 and the initial survey methods for stage 2 are described here.

### Stage 1: Creation of an Exhaustive List of Outcomes and a Questionnaire Survey

#### Creation of an Exhaustive List of Outcomes

All possible outcomes of bariatric surgery were identified from systematic literature reviews of studies reporting clinical and patient-reported outcomes (PROs) [1, 2]. These were supplemented with additional outcomes identified from qualitative research studies of bariatric surgery and qualitative interviews with patients who had undergone surgery [16, 17].

**Fig. 1:**
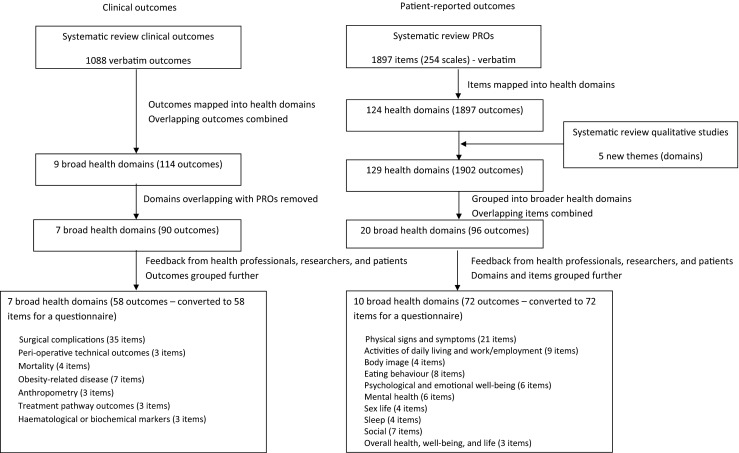
Identifying and grouping outcomes of bariatric surgery into domains for a questionnaire study

Systematic reviews identified 1088 clinical outcomes, and 68 validated PRO measures (PROMs) [1, 2]. A full version of each PROM was obtained and all scales and items (1897 individual items) listed electronically. The clinical outcomes were also listed, creating two lists of outcomes (clinical and PROs). These lists were scrutinised and categorised into health domains according to the method described by Macefield et al. [18]. Briefly, each outcome was assigned a domain independently by at least two researchers who included expert health professional researchers (surgeons, a dietitian, a specialist nurse, a health psychologist) and methodologists. Domains were generated until saturation, or all outcomes were mapped to a health domain. Outcomes which overlapped or were synonyms of each other were combined. Differences between researchers were resolved by discussion within the study team and with the senior author (JB). The PRO list was supplemented with additional domains extracted from a review of qualitative research studies investigating the patient’s perspective of bariatric surgery [16, 17]. This identified five themes which were new and different to the list of PRO domains (‘the development of new addictions after bariatric surgery’, ‘excess skin’, ‘stigma’, ‘personal identity’, and ‘normality’) [16, 17]. Semi-structured interviews conducted with a purposeful sample of seven bariatric surgery patients were undertaken to elicit additional themes relating to outcomes of bariatric surgery. However, these did not yield any new themes that had not already been reported in the literature. After the final feedback from expert health professionals, researchers, and patient research partners, seven broad clinical domains (85 outcomes) and ten PRO domains (72 outcomes) were created. These included the following: surgical complications; perioperative technical outcomes; mortality; obesity-related disease; anthropometry; treatment pathway outcomes; haematological or biochemical markers; physical signs and symptoms; activities of daily living and work/employment; body image; eating behaviour; psychological and emotional well-being; mental health; sex life; sleep; social; and overall health, well-being, and life [1, 2, 16, 17]. The full process is outlined in Fig. 1.

#### Development of a Questionnaire Survey

**Fig. 2:**
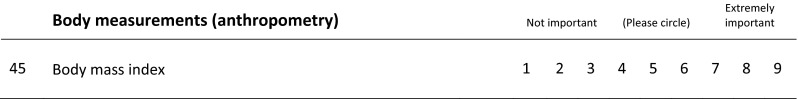
An example questionnaire item

The seven clinical health domains (58 outcomes) and ten PRO health domains (72 outcomes) were developed into a 130-item questionnaire presented in four sections: (1) Short- and long-term complications of surgery; (2) Clinical effectiveness of surgery; (3) Physical signs, symptoms, and other measures (including haematological/biochemical markers and treatment pathway outcomes); and (4) Impact of surgery on quality of life and well-being. A questionnaire item was written for each of the 58 clinical outcomes and 72 PROs by two members of the research team. The wording and layout of the questionnaire underwent minor modification to ensure face validity through members of the research team and two patient research partners reading iterative versions until it was acceptable and understandable. For items in sections Introduction to Stage 1: Creation of an Exhaustive List of Outcomes and a Questionnaire Survey of the questionnaire, the technical terms were also included in brackets next to the patient-approved wording where appropriate (for both the professional and patient questionnaire). The full wording used for each item can be found in the questionnaires which are included as supplementary files. All questionnaires asked participants to rate the importance of each item (outcome) on a scale of 1 (not important) to 9 (extremely important) to be retained in a core outcome set. An example questionnaire item is presented in Fig. 2. Basic demographic data were collected in the questionnaire.

### Stage 2: Survey of Health Professionals and Patients

The questionnaire was used to survey the views of specialist bariatric surgery health professionals (surgeons, nurses, dietitians, psychologists, physicians, and anaesthetists) and patients who had undergone surgery. There is no specific guidance for determining the sample size required for Delphi surveys. For this study, a sample of 100 professionals and 100 patients was anticipated to provide a representative sample including a broad set of views.

All professional members of the British Obesity and Metabolic Surgery Society (BOMSS) were sent an invitation and questionnaire in the post. Additionally, professionals from the Association of Physicians Specialising in Obesity UK (APSO-UK), the Society for Obesity and Bariatric Anaesthesia (SOBA), the British Psychological Society (BPS), and an informal e-mail list of clinical psychologists working in bariatric surgery in the UK were sent an e-mail invite to participate in the survey. Professionals who replied back to the research team that they were interested in participating were sent the questionnaire via post. Additionally, health professionals participating in a large UK-based bariatric surgery trial [15] were given the questionnaire at a trial meeting. An e-mail reminder was sent to health professionals 1 month after sending the questionnaire to encourage a response.

Patients were identified from two hospital databases in the south of England. A purposeful sample received an invitation to participate, sampled for gender, type of surgery, and time since surgery, in order to obtain as wide a variety of these characteristics as possible. The questionnaire survey was posted to patients that returned a reply slip and signed consent form. Patients who had consented to participate but had not returned their questionnaire after 1 month were sent a reminder letter with a second copy of the questionnaire to encourage a response. Full ethical approval for the study was obtained from Southwest-Frenchay Research Ethics Committee (reference 11/SW/0248).

### Analysis of Questionnaire Responses

The percentage of participants within each stakeholder group (professionals and patients) rating each item an 8 or 9 was calculated. Items were then ranked. An item was classed as ‘very important’ (or prioritised) if at least 70 % of participants rated it 8 or 9. These criteria were chosen within our research team and based on feedback from health professionals completing the survey, as no accepted methods are available for analyses of Delphi surveys. The number and percentage of items prioritised by both groups (overlapping items) and by one group only (discordant items) were calculated. This was calculated for all 130 items in the questionnaire and also for each of the four sections of the questionnaire (complications of surgery; clinical effectiveness; physical signs, symptoms, and other measures; quality of life and well-being), in order to examine the *types* of items prioritised by each group. Interrater agreement of items prioritised or not prioritised was assessed using the kappa statistic (*ĸ*) [19]. The level of agreement was classified as poor (*ĸ* ≤ 0.0), slight (*ĸ* 0.01–0.20), fair (*ĸ* 0.21–0.40), moderate (*ĸ* 0.41–0.60), substantial (*ĸ* 0.61–0.80), and almost perfect (*ĸ* 0.81–0.99) [19]. The percentage of prioritised items within each of the four sections of the questionnaire was also calculated for (i) surgeons, (ii) dietitians, (iii) specialist nurses, and (iv) other professionals (including bariatric physicians, psychologists, anaesthetists, GPs, and physiotherapists); however, agreement between health professional sub-groups was not formally tested using kappa statistics. All statistical analyses were carried out using STATA 13 statistical software [20].

## Results

Health professional and patient response rates were 36.6 % (168/459) and 19.4 % (90/465), respectively. Participant characteristics are presented in Table 1.

**Table 1:** Characteristics of health professionals and patients participating in the survey (*n* = 258)

Health professionals (*n* = 168)	
Number female (%)	77 (45.8)
Country (%)	
UK	160 (95.2)
Republic of Ireland	2 (1.2)
Belgium	1 (0.6)
Not specified	5 (3.0)
Type of health professional (%)	
Surgeon	81 (48.2)
Dietitian	33 (19.6)
Specialist nurse	24 (14.3)
Bariatric physician	12 (7.1)
Psychologist	10 (6.0)
Anaesthetist	3 (1.8)
GP	3 (1.8)
Physiotherapist	1 (0.6)
Others	1 (0.6)
Patients (*n* = 90)	
Number female (%)	59 (65.6)
Mean age (SD)	54.4 years (9.6 years)
Number with ‘White British’ ethnicity (%)	86 (95.6)
Marital status (%)	
Married	52 (57.8)
Divorced	18 (20.0)
Single	14 (15.6)
Co-habiting	3 (3.3)
Widowed	3 (3.3)
Employment status (%)	
Employed full-time	34 (37.8)
Retired	21 (23.3)
Employed part-time	11 (12.2)
Unemployed and seeking work	8 (8.9)
Unemployed on sickness/disability	7 (7.8)
Housewife/househusband	5 (5.6)
Others	4 (4.4)
Number post-operative (%)	88 (97.8)
Type of operation (%)	
Roux-en-Y gastric bypass	58 (65.9)
Adjustable gastric band	21 (23.9)
Sleeve gastrectomy	6 (6.8)
More than one type of surgery	2 (2.3)
Others	1 (1.1)
Mean time since surgery (SD)^a^	3.5 years (2.1 years)

*SD* standard deviation

^a^This data was missing for five post-operative patients, making *n* = 83

### Differences in Stakeholder Views

#### Professionals Versus Patients

Items rated 8–9 by 70 % of professionals or patients (‘very important’ or prioritised items) are presented in Table 2. The top ranked items were ‘diabetes’ for professionals (89.8 % rated 8–9) and ‘leakage from bowel joins within the abdomen’ for patients (86.5 % rated 8–9). Professionals and patients prioritised 18 and 25 items, respectively. There were ten items (out of 130, 7.7 %) common to both, which included diabetes and surgical complications such as ‘risk of death within a month of surgery’ and leakage from bowel joins within the abdomen (Tables 2 and 3). However, 23 (17.7 %) items were discordant or prioritised by one group only (eight for professionals, 15 for patients) such as ‘weight’ and ‘re-admission rates’ (professionals only) and ‘feeling able to live a normal life’ and ‘excess skin following weight loss’ (patients only) (Tables 2 and 3). The kappa statistic of 0.363 indicates a fair level of agreement on the items prioritised between professionals and patients.

**Table 2:** Items prioritised (rated 8–9 by ≥70 % of participants) by professionals and patients^a^

Items rated 8–9 by ≥70 % of both professionals and patients (10)	% professionals rating 8–9 (*n* = 168)	% patients rating 8–9 (*n* = 90)
Improvement in diabetes, diabetes no longer being present, or a reduction in diabetic medication (measure of diabetes, e.g. HbA1c)	89.8	82.2
Risk of death during the operation (perioperative mortality)	86.7	84.1
Risk of death within a month of surgery, in hospital or at home (≤30 day mortality)	86.1	83.0
Risk of death from surgical complications whilst still in hospital (in hospital mortality)	85.5	83.0
Leaking of bowel contents into the abdomen through a hole where the bowel is joined or stapled (anastomotic leak)	84.1	86.5
The gastric band eroding/growing into the stomach (band moves from outside to the inside of stomach) leading to the need for further surgery (band erosion)	79.4	78.2
Leaking of stomach contents through a hole in the stomach (gastric fistula)	79.3	82.0
The band slipping out of place and needing more surgery to correct it (band slippage)	78.2	75.6
Risk of death more than a month after surgery, in hospital or at home (>30 day mortality)	74.1	80.7
Whole body infection which requires prolonged admission to hospital (septicaemia)	70.5	83.1
Items rated 8–9 by ≥70 % of health professionals only (8)		
A measurement of weight	87.4	58.4
Being able to breathe easily when sleeping/using a sleep mask less (obstructive sleep apnoea)	80.2	65.2
Body mass index (BMI)	76.6	49.4
Unexpected return to hospital for unplanned procedures or urgent review (re-admission rates)	76.5	51.7
Reduction/lowering of blood pressure to a healthy level, or a reduction in blood pressure medication (hypertension)	76.0	69.7
Twisting or abnormal movement of the bowel or intestines, which can cause blockages, pain or nausea and may need additional surgery (internal hernia)	74.5	66.7
Infection of the gastric band (band infection)	71.5	66.7
Being able to accomplish work tasks, or to take up work/paid employment	70.1	59.6
Items rated 8–9 by ≥70 % of patients only (15)		
Stroke (cerebrovascular accident) (complication of surgery)	47.6	83.0
Kidney failure (renal failure) (complication of surgery)	47.0	80.5
Abnormal narrowing of the bowel caused by scar tissue or stapling, which might cause a blockage (stenosis)	62.8	76.4
Being able to stop eating when feeling full	61.4	76.4
Normality (feeling able to live a ‘normal’ life)	54.5	76.4
Blood clot in the leg or lung (venous thromboembolism) (complication of surgery)	64.0	76.1
Having a positive outlook on life and expectations for the future	53.3	75.3
Heart’s blood supply is blocked, or interrupted, by a build-up of fatty substances in the heart’s arteries (ischaemic/coronary heart disease (complication of surgery)	41.5	73.6
Bleeding from the internal bowel staples (staple line bleed)	56.2	73.0
Feeling in control of health and well-being	47.2	72.7
Ulcers developing at the new join between the two pieces of bowel (anastomotic ulceration)	56.1	71.9
Mobility (e.g., being able to walk, climb stairs, bend, cross legs, get up from chairs)	65.9	71.9
Having a healthy/balanced eating pattern	59.0	71.9
Reduction in the chance of having heart problems in the future (adjusted cardiovascular risk)	59.9	71.1
Excess skin or skin folds following weight loss	46.7	70.8

^a^Wording of items shown is the exact wording from the questionnaire

**Table 3:** A comparison of items prioritised (rated 8–9 by ≥70 % of participants) within each section of the questionnaire, by professionals and patients

Section of questionnaire	Items prioritised (%)					
	Both HCPs and patients	Neither	HCPs only	Patients only	Discordant items^a^	Kappa statistic (*ĸ*)
Complications of surgery (42 items)	9 (21.4)	24 (57.1)	2 (4.8)	7 (16.7)	9 (21.5)	0.517
Clinical effectiveness (10 items)	1 (10.0)	4 (40.0)	4 (40.0)	1 (10.0)	5 (50.0)	0.000
Signs, symptoms, and other measures (27 items)	0 (0.0)	26 (96.3)	1 (3.7)	0 (0.0)	1 (3.7)	0.000
Quality of life and well-being (51 items)	0 (0.0)	43 (84.3)	1 (2.0)	7 (13.7)	8 (15.7)	−0.036
All items (130)	10 (7.7)	97 (74.6)	8 (6.2)	15 (11.5)	23 (17.7)	0.363

*HCPs* healthcare professionals

^a^Calculated as number of items prioritised by HCPs only + number of items prioritised by patients only

There was moderate agreement (*ĸ* 0.517) on items prioritised in the complications of surgery section, with nine (out of 42, 21.4 %) items prioritised by both groups and nine (21.4 %) items prioritised by one group only (two by professionals and seven by patients). A poor level of agreement (*ĸ* −0.036) was found for the quality of life and well-being section, with no items prioritised by both groups and eight (out of 51, 15.7 %) prioritised by one group only (one by professionals, seven by patients). In the clinical effectiveness section, one item (out of 10, 10.0 %) was prioritised by both groups and five (50.0 %) prioritised by one group only (four by professionals, one by patients) (*ĸ* 0.000). In the signs, symptoms, and other measures section, only one item was prioritised by professionals (re-admission rates) and none by patients (*ĸ* 0.000).

#### Health Professional Sub-Groups

Table 4 shows the number and percentage of items prioritised in each section of the questionnaire, by health professional sub-group, although these were not compared statistically. Overall, nurses prioritised the greatest number of items in the questionnaire (33 out of 130, 25.4 %). ‘Other professionals’ prioritised the least complications of surgery (11.9 versus 26.2 % for nurses) and clinical effectiveness items (40.0 versus 70.0 % for nurses) and surgeons the least quality of life and well-being items (2.0 versus 21.6 % for nurses). Within the signs, symptoms, and other measures section, all professionals prioritised re-admission rates, except dietitians. Also within this section, ‘vitamin levels’ and ‘mineral levels’ were prioritised by dietitians and nurses only and ‘dysphagia’ by nurses only.

**Table 4:** Items prioritised (rated 8–9 by ≥70 % of participants) within each section of the questionnaire, by health professional sub-group

Section of questionnaire	Items prioritised by health professional sub-group (%)			
	Surgeons (*n* = 81)	Dietitians (*n* = 33)	Nurses (*n* = 24)	Other professionals^a^ (*n* = 30)
Complications of surgery (42 items)	11 (26.2)	10 (23.8)	11 (26.2)	5 (11.9)
Clinical effectiveness (10 items)	5 (50.0)	5 (50.0)	7 (70.0)	4 (40.0)
Signs, symptoms, and other measures (27 items)	1 (3.7)	2 (7.4)	4 (14.8)	1 (3.7)
Quality of life and well-being (51 items)	1 (2.0)	4 (7.8)	11 (21.6)	6 (11.8)
All items (130)	18 (13.8)	21 (16.2)	33 (25.4)	16 (12.3)

^a^Includes bariatric physicians, psychologists, anaesthetists, GPs, physiotherapists, and other health professionals

## Discussion

This study investigated and compared how health professionals and patients prioritised outcomes of bariatric surgery. Overall, 33 different outcomes were prioritised by either health professionals and/or patients, with diabetes and leakage from bowel joins within the abdomen considered the most important outcomes for professionals and patients, respectively. Although there was some agreement of outcomes prioritised (10 of the 33 different outcomes prioritised overlapped between the two groups), the remaining 23 outcomes were prioritised by one group only, highlighting important differences between professionals and patients. Patients prioritised seven quality of life items, none of which were prioritised by professionals. Agreement was better for items prioritised in the complications of surgery section, with nine of these items overlapping between professionals and patients. Health professional subgroups were also examined which showed that only dietitians and nurses prioritised nutritional outcomes, and surgeons prioritised only one quality of life outcome (versus four to 11 in the other sub-groups). In view of the different opinions of the professionals and patients, it is recommended that the views of both patients and all relevant health professionals are considered when deciding which outcomes to measure to evaluate bariatric surgery.

Differences between patients’ and professionals’ views of important outcomes have been noted in other disease areas, with patients placing more importance on outcomes relating to quality of life, particularly psychological and emotional well-being, than professionals [3–9]. For example, in a cross-sectional questionnaire study where clinicians and multiple sclerosis patients were asked which of the scales in the Short Form-36 Health-Related Quality of Life (HRQL) questionnaire were most important to them, clinicians prioritised physical function and physical role limitations, whereas patients prioritised mental health and emotional role limitations [3]. Additionally, a cross-sectional questionnaire study in rheumatology in which patients and clinicians were asked to concurrently rank a list of outcomes according to their importance found that clinicians were not often able to accurately identify the outcomes that were most important to their patients, particularly mental functioning outcomes [4]. Further research with rheumatoid arthritis patients identified important outcomes that were not being measured to assess treatment efficacy (e.g. wellness, return to normality, and fatigue) [5, 6]. A systematic review which examined agreement in HRQL scores between physicians and patients, mainly in childhood cancer, found less agreement in ratings of subjective domains of HRQL (such as emotion, cognition, and pain) but better agreement in objective domains such as mobility [7]. Most recently, in developing a core outcome set for breast reconstruction surgery, professionals prioritised certain operation-specific complications which patients did not, whereas patients prioritised ‘self-esteem’, ‘emotional well-being’, and ‘physical well-being’, which professionals did not [8]. In developing a core information set for oesophageal cancer, Blazeby et al. found that patients rated information about longer-term quality of life outcomes more highly, whereas professionals rated information about shorter-term clinical outcomes more highly [9]. Taken together, the literature supports our study’s findings that patients prioritise more quality of life outcomes than professionals and that professionals place more emphasis on clinical outcomes and readmission rates. This is understandable given the need for patients to adapt their day-to-day lives in the long term after surgery.

Another noteworthy finding within the health professional sub-groups was that dietitians and nurses prioritised nutritional outcomes and physical symptoms associated with eating, which surgeons and other professionals did not. Surgeons also prioritised only one quality of life outcome, whereas the other professional sub-groups prioritised between 4 and 11. This is understandable as the focus of care provided by allied health professionals and nurses is to support bariatric surgery patients with these types of clinical and quality of life issues. To our knowledge, how different health professionals prioritise different outcomes of treatments has not been investigated previously and warrants further investigation with larger numbers of professionals.

A strength of this study is that it is novel, and a variety of health professionals and patients have been surveyed. However, there are some limitations which should be acknowledged. Response rates could have been better. There is always the risk of non-response bias with low response rates, meaning the responses received may not be representative of the whole population including non-responders [21]. We predicted that response rates would be low based on response rates to similar surveys carried out within our research team, and as such invited large numbers of health professionals and patients in order to get as close to 100 of each as possible who completed the survey. This study was based in the UK only, although a few professionals from outside the UK participated. The patients were recruited from two centres in the south of England and were primarily Caucasian (96 %). This reflects the primarily Caucasian ethnicity of patients who undergo bariatric surgery in the UK (83 %) [22]; however, we appreciate that this does not reflect the ethnicity of patients undergoing bariatric surgery in other countries, particularly Asia and Latin America. Once the core outcome set has been developed, future work will be undertaken to validate it internationally. The majority of our patient sample was female (65.6 %), which reflects the much higher prevalence of women undergoing bariatric surgery than men reported around the world (75–79 %) [22–24]. The mean age of our patient participants was 54.4 years (SD 9.6 years). In the UK, the majority of bariatric surgery operations are carried out in individuals aged 35–54, and in other countries, the mean age of bariatric surgery patients has been reported to be 44–45 years of age [22–24]. Another strength of our study was that our patient participants had a mean time since surgery of 3.5 years, which meant they had experience of living with the outcomes of surgery in the long term after the initial ‘honeymoon’ phase had worn off. Future studies could aim to seek the views of more pre-operative patients awaiting bariatric surgery as it is possible that outcomes prioritised may be different at the pre-operative stage compared with the post-operative stage.

Taken together, the literature, as well as our study, highlights the critical importance of including both patients’ and professionals’ views when deciding which outcomes to measure to evaluate the effectiveness of treatments, particularly treatments for chronic diseases such as obesity. Our study indicates that different health professionals may have different views of important outcomes of bariatric surgery. A multi-disciplinary team is typically involved in the care of obese patients; therefore, it is crucial that the views of all relevant health professionals and patients are taken into account when deciding what outcomes are most important to evaluate bariatric surgery [25, 26]. The research presented is part of an overall project to develop a core outcome set for bariatric surgery (the BARIACT project) [15]. The final stages of the project include two more questionnaire rounds, followed by consensus meetings with patients and professionals, where the final core outcome set will be agreed. Use of the core outcome set will help to standardise the data resulting from future bariatric surgery trials, meaning that trial data is more easily synthesised in meta-analyses to better inform future clinical practice. It will also help to ensure that the outcomes most important to all relevant stakeholders are used to evaluate bariatric surgery.

## Electronic supplementary material

Below is the link to the electronic supplementary material.

ESM 1 (PDF 543 kb)

ESM 2 (PDF 219 kb)
