# Supplementary material for: A Comparison of Health Professionals’ and Patients’ Views of the Importance of Outcomes of Bariatric Surgery
Source: Obes Surg. 2016 May 2;26(11):2738–46. doi: 10.1007/s11695-016-2186-0 (PMC5069338; doi:10.1007/s11695-016-2186-0)
Supplement: Supplementary file 1 — (PDF 543 kb) [file 11695_2016_2186_MOESM1_ESM.pdf]

Developing a core outcome set for studies in weight loss surgery

Round one questionnaire

ID

Please answer all the questions yourself. The information that you provide will remain anonymous.

When you have completed the questionnaire, please return it in the stamped addressed envelope.

ID

SECTION 1 First, a few things about you

To allow us to analyse the results of the study, we need some brief information about you and the type of surgery you and your institution perform. Please tick the most appropriate box(es). Preferably please provide information for your main institution of employment, or for yourself if you work in a single handed practice.

1. **Your initials:**..

2. **Your gender:** Male ☐ Female ☐

3. **In which country do you work?** ....

4. **Your job / role on the bariatric MDT (tick all that apply):**

   Bariatric Surgeon ☐
   Bariatric Physician ☐
   Anaesthetist ☐
   Specialist Nurse ☐
   Dietician ☐
   Psychologist ☐
   Trainee ☐
   Other ☐
   please specify…………

5. **How many bariatric procedures did you or your institution perform in 2013?**

   1 -50 ☐
   50 - 100 ☐
   100 - 200 ☐
   200 – 300 ☐
   300 - 500 ☐
   > 500 ☐
   Unknown ☐

Attach name and address sticker here

ID

SECTION 2 Short and long term complications

This section lists events that may occur during, or after, weight loss surgery. Please note, these are only possibilities and do not occur in everyone. The words in brackets are the medical terminology. Please rate how important you think it is that the following events are measured in studies of weight loss surgery and circle the number that best represents your opinion.

		Not important			(Please circle)					Extremely important
	**For example**: Pain or discomfort in stomach area	1	2	3	4	5 (circled)	6	7	8	9

		Not important			(Please circle)					Extremely important
	**Complications that might occur during the operation**									
1	Bleeding problems, such as a collection of blood or loss of blood during the operation (peri- operative haemorrhage)	1	2	3	4	5	6	7	8	9
2	The risk of accidentally damaging other organs during the operation (organ injury)	1	2	3	4	5	6	7	8	9
3	The likelihood of needing to use a large cut/incision during surgery, rather than planned keyhole surgery (conversion to open)	1	2	3	4	5	6	7	8	9
	**Infection**									
4	Infection inside the body where the operation was carried out (deep abscess)	1	2	3	4	5	6	7	8	9
5	Problems with the wound such as infection, oozing, or failure to heal properly (wound infection or dehiscence)	1	2	3	4	5	6	7	8	9
6	Whole body infection which requires prolonged admission to hospital (septicaemia)	1	2	3	4	5	6	7	8	9
	**Haemorrhage**									
7	Bleeding problems in stomach or bowel which may cause blood to be seen in bowel movements (gastrointestinal bleeding)	1	2	3	4	5	6	7	8	9

ID

		Not important				(Please circle)				Extremely important
8	Bleeding inside the abdomen where the operation was carried out (intra-abdominal bleeding)	1	2	3	4	5	6	7	8	9
9	Bleeding from the wound (wound bleeding / port site haematoma)	1	2	3	4	5	6	7	8	9
	**Surgical joins between internal organs (anastomosis)**									
10	Leaking of stomach contents through a hole in the stomach (gastric fistula)	1	2	3	4	5	6	7	8	9
11	Leaking of bowel contents into the abdomen through a hole where the bowel is joined or stapled (anastomotic leak)	1	2	3	4	5	6	7	8	9
12	Abnormal narrowing of the bowel caused by scar tissue or stapling, which might cause a blockage (stenosis)	1	2	3	4	5	6	7	8	9
13	Bleeding from the internal bowel staples (staple line bleed)	1	2	3	4	5	6	7	8	9
14	Ulcers developing at the new join between the two pieces of bowel (anastomotic ulceration)	1	2	3	4	5	6	7	8	9
	**Band related complications**									
15	Infection of the gastric band (band infection)	1	2	3	4	5	6	7	8	9
16	Problems with the port, such as flipping or needing the port to be relocated (port erosion or revisions)	1	2	3	4	5	6	7	8	9
17	Infection of the port which is used to change the band size (port infection)	1	2	3	4	5	6	7	8	9
18	The gastric band eroding/growing into the stomach (band moves from outside to the inside of stomach) leading to the need for further surgery (band erosion)	1	2	3	4	5	6	7	8	9
19	The band slipping out of place and needing more surgery to correct it (band slippage)	1	2	3	4	5	6	7	8	9
20	Intolerance of the band	1	2	3	4	5	6	7	8	9
21	Pain in the stomach caused by overeating/too much food stretching the stomach above the band (pouch dilation)	1	2	3	4	5	6	7	8	9
22	Damage to the band, which might require band replacement (Iatrogenic injury [device])	1	2	3	4	5	6	7	8	9
23	Failure of the port or tube to work properly (port malfunction)	1	2	3	4	5	6	7	8	9

		Not important			(Please circle)			Extremely important		
24	The need for further surgery to make changes to the band (band revisions)	1	2	3	4	5	6	7	8	9
	**Obstruction / hernia**									
25	Twisting or abnormal movement of the bowel or intestines, which can cause blockages, pain or nausea and may need additional surgery (internal hernia)	1	2	3	4	5	6	7	8	9
26	A lump or bulge in the skin through the scar where the surgery was performed (external hernia, incisional)	1	2	3	4	5	6	7	8	9
27	Build up of scar tissue in the bowel causing obstruction or abdominal pain (adhesional obstruction)	1	2	3	4	5	6	7	8	9
28	Vomiting or excessive bloating after the operation whilst still in hospital (ileus)	1	2	3	4	5	6	7	8	9
	**General complications of surgery**									
29	One or more areas of the lungs collapsing or not inflating properly (atelectasis)	1	2	3	4	5	6	7	8	9
30	Needing a machine to help with breathing (ventilation)	1	2	3	4	5	6	7	8	9
31	Chest infection (lower respiratory tract infection)	1	2	3	4	5	6	7	8	9
32	Heart's blood supply is blocked, or interrupted, by a build-up of fatty substances in the heart's arteries (ischaemic/coronary heart disease)	1	2	3	4	5	6	7	8	9
33	Irregular or abnormal heart beat (arrhythmia)	1	2	3	4	5	6	7	8	9
34	Blood clot in the leg or lung (venous thromboembolism)	1	2	3	4	5	6	7	8	9
35	Stroke (cerebrovascular accident)	1	2	3	4	5	6	7	8	9
36	Kidney failure (renal failure)	1	2	3	4	5	6	7	8	9
37	Bladder infection in 'wee/pee' (Urinary tract infection)	1	2	3	4	5	6	7	8	9
38	Unintentional passing of urine (incontinence)	1	2	3	4	5	6	7	8	9

ID

		Not important			(Please circle)			Extremely important		
	**Mortality**									
39	Risk of death during the operation (peri-operative mortality)	1	2	3	4	5	6	7	8	9
40	Risk of death from surgical complications whilst still in hospital (in hospital mortality)	1	2	3	4	5	6	7	8	9
41	Risk of death within a month of surgery, in hospital or at home (≤30 day mortality)	1	2	3	4	5	6	7	8	9
42	Risk of death more than a month after surgery, in hospital or at home (>30 day mortality)	1	2	3	4	5	6	7	8	9

ID

SECTION 3 Effectiveness of weight loss surgery

The following section lists different ways to measure the effectiveness of weight loss surgery. Please rate how <u>important</u> you think it is that the following information is collected to <u>measure the impact/effectiveness</u> of the surgery and circle the number that represents your opinion. The words in brackets are the medical terminology.

		Not important				(Please circle)				Extremely important
	**Body measurements (anthropometry)**									
43	A measurement of weight	1	2	3	4	5	6	7	8	9
44	Body dimensions such as waist and hip measurements	1	2	3	4	5	6	7	8	9
45	Body mass index	1	2	3	4	5	6	7	8	9
	**Obesity related disease**									
46	Improvement in abnormal or irregular heartbeat (arrhythmia)	1	2	3	4	5	6	7	8	9
47	Reduction/lowering of blood pressure to a healthy level, or a reduction in blood pressure medication (hypertension)	1	2	3	4	5	6	7	8	9
48	Reduction in the chance of having heart problems in the future (adjusted cardiovascular risk)	1	2	3	4	5	6	7	8	9
49	Improvement in diabetes, diabetes no longer being present, or a reduction in diabetic medication (measure of diabetes e.g. HbA1c)	1	2	3	4	5	6	7	8	9
50	Reduction in the amount of fat and cholesterol in the blood, or a reduction in medication (measure of dyslipidaemia)	1	2	3	4	5	6	7	8	9
51	Being able to breathe easily when sleeping / using a sleep mask less (obstructive sleep apnoea)	1	2	3	4	5	6	7	8	9
52	Improvement in joint disease	1	2	3	4	5	6	7	8	9

ID

SECTION 4 Physical signs, symptoms and other measures

The following section lists some physical signs and symptoms that some people may experience before or after weight loss surgery. It also lists some measurements that may be taken. Some of these signs and symptoms may get better after surgery. Some may get worse, or remain the same. Please note, these are only possibilities and do not occur in everyone. The words in brackets are the medical terminology. These are all measured by health professionals, and are not self-reported by the patient.

Please rate how important you think it is that the following symptoms are measured in research studies of weight loss surgery, and circle the number that best represents your opinion.

		Not important				(Please circle)				Extremely important
	**Signs**									
53	Hair loss	1	2	3	4	5	6	7	8	9
54	Problems hearing	1	2	3	4	5	6	7	8	9
55	Problems with gums or teeth	1	2	3	4	5	6	7	8	9
56	Problems with vision	1	2	3	4	5	6	7	8	9
57	Swelling or retaining water (oedema)	1	2	3	4	5	6	7	8	9
58	Skin problems or irritations (such as rashes, sores or loose skin, ulcers)	1	2	3	4	5	6	7	8	9
59	Loss of sensation in hands and feet (peripheral neuropathy/paraesthesia)	1	2	3	4	5	6	7	8	9
	**Symptoms**									
60	Feeling out of breath (breathlessness)	1	2	3	4	5	6	7	8	9
61	Belching, bloating, or gas (flatulence)	1	2	3	4	5	6	7	8	9
62	Constipation or difficulty passing stool	1	2	3	4	5	6	7	8	9
63	Diarrhoea or loose bowel motion	1	2	3	4	5	6	7	8	9

ID

		Not important			(Please circle)					Extremely important
64	Pain or discomfort in the body	1	2	3	4	5	6	7	8	9
65	Feeling hot or sweaty	1	2	3	4	5	6	7	8	9
66	Feeling light-headed or dizzy	1	2	3	4	5	6	7	8	9
67	Feeling sick or vomiting (nausea)	1	2	3	4	5	6	7	8	9
68	Numbness or tingling in the body	1	2	3	4	5	6	7	8	9
69	Heartburn or acid indigestion (reflux)	1	2	3	4	5	6	7	8	9
70	Problems swallowing or bringing food back up (dysphagia/regurgitation)	1	2	3	4	5	6	7	8	9
71	Problems controlling the bladder (urinary incontinence)	1	2	3	4	5	6	7	8	9
72	Appearing physically tired or lacking in energy (physical fatigue)	1	2	3	4	5	6	7	8	9
73	Pain or discomfort in stomach area	1	2	3	4	5	6	7	8	9
	**Other Measures**									
74	A measurement of vitamin levels	1	2	3	4	5	6	7	8	9
75	How many calories patients consume (energy intake)	1	2	3	4	5	6	7	8	9
76	A measurement of mineral levels	1	2	3	4	5	6	7	8	9
77	The length of time spent in hospital after admission for surgery (length of hospital stay)	1	2	3	4	5	6	7	8	9
78	How long the operation takes (operative time)	1	2	3	4	5	6	7	8	9
79	Unexpected return to hospital for unplanned procedures or urgent review (re-admission rates)	1	2	3	4	5	6	7	8	9

ID

SECTION 5 Issues relating to quality of life and wellbeing after surgery

The following section lists some other areas of life that can be affected by having weight loss surgery. These issues are all patient reported, i.e. issues that are reported directly from the patient themselves and are not interpreted by clinicians or anyone else.

Please rate how important you think it is, that the following issues are measured in research studies of weight loss surgery, and circle the number that best represents your opinion.

		Not important				(Please circle)				Extremely important
	**Activities of daily living and work/employment**									
80	Being able to carry out usual activities (not related to paid employment) such as personal hygiene, housework, managing finances	1	2	3	4	5	6	7	8	9
81	Being able to shop for clothes that fit	1	2	3	4	5	6	7	8	9
82	Being able to fit into spaces in public places (e.g. fit into seats or through aisles)	1	2	3	4	5	6	7	8	9
83	Mobility (e.g. being able to walk, climb stairs, bend, cross legs, get up from chairs)	1	2	3	4	5	6	7	8	9
84	Fitness (strength and endurance)	1	2	3	4	5	6	7	8	9
85	Being able to participate in, and enjoy physical activities	1	2	3	4	5	6	7	8	9
86	Being able to accomplish work tasks, or to take up work/paid employment	1	2	3	4	5	6	7	8	9
87	Satisfaction and recognition at work (if in paid employment)	1	2	3	4	5	6	7	8	9
88	Relationships with work colleagues (if in paid employment)	1	2	3	4	5	6	7	8	9
	**Body image**									
89	Feeling satisfied and confident with one’s body	1	2	3	4	5	6	7	8	9
90	Feeling in control of weight and appearance	1	2	3	4	5	6	7	8	9
91	Feeling like the mind and body are in tune with each other	1	2	3	4	5	6	7	8	9

ID

		Not important			(Please circle)					Extremely important
92	Excess skin or skin folds following weight loss	1	2	3	4	5	6	7	8	9
	**Eating behaviour**									
93	Having a healthy/balanced eating pattern	1	2	3	4	5	6	7	8	9
94	Being able to recognise hunger feelings	1	2	3	4	5	6	7	8	9
95	Being able to stop eating when feeling full	1	2	3	4	5	6	7	8	9
96	Time spent thinking about food	1	2	3	4	5	6	7	8	9
97	Eating for emotional reasons	1	2	3	4	5	6	7	8	9
98	Feeling guilty or upset after eating	1	2	3	4	5	6	7	8	9
99	Eating in secret	1	2	3	4	5	6	7	8	9
100	Eating differently in social situations than normal	1	2	3	4	5	6	7	8	9
	**Psychological and emotional wellbeing**									
101	How individuals feel others perceive them	1	2	3	4	5	6	7	8	9
102	Self-esteem and self-confidence (how someone perceives themself)	1	2	3	4	5	6	7	8	9
103	Mood swings	1	2	3	4	5	6	7	8	9
104	Stress levels	1	2	3	4	5	6	7	8	9
105	Coping (how someone feels with stress or difficulties)	1	2	3	4	5	6	7	8	9
106	Feeling in control of emotional or psychological wellbeing	1	2	3	4	5	6	7	8	9
	**Mental health**									
107	Depression	1	2	3	4	5	6	7	8	9
108	Anxiety	1	2	3	4	5	6	7	8	9
109	Hostility (level of anger, and ability to manage this)	1	2	3	4	5	6	7	8	9

		Not important				(Please circle)				Extremely important
110	Fears or phobias	1	2	3	4	5	6	7	8	9
111	Suicidal thoughts	1	2	3	4	5	6	7	8	9
112	Other addictive behaviours (e.g. alcohol, drugs, gambling, shopping)	1	2	3	4	5	6	7	8	9
	**Sex life**	1	2	3	4	5	6	7	8	9
113	Interest in and enjoyment of sex	1	2	3	4	5	6	7	8	9
114	Ability to physically participate in sex	1	2	3	4	5	6	7	8	9
115	Sexual confidence	1	2	3	4	5	6	7	8	9
116	Sexual attention from others	1	2	3	4	5	6	7	8	9
	**Sleep**									
117	Ability to fall asleep at night	1	2	3	4	5	6	7	8	9
118	Overall quality of sleep	1	2	3	4	5	6	7	8	9
119	Sleepiness during the day	1	2	3	4	5	6	7	8	9
120	Snoring which affects others	1	2	3	4	5	6	7	8	9
	**Social**	1	2	3	4	5	6	7	8	9
121	Relationship with partner/spouse	1	2	3	4	5	6	7	8	9
122	Relationship with, and/or ability to care for, children	1	2	3	4	5	6	7	8	9
123	Relationship with other family members	1	2	3	4	5	6	7	8	9
124	Relationship with friends	1	2	3	4	5	6	7	8	9
125	Treatment from people in wider society	1	2	3	4	5	6	7	8	9
126	Having confidence to participate in social activities	1	2	3	4	5	6	7	8	9
127	Feeling in control of social life	1	2	3	4	5	6	7	8	9

		Not important				(Please circle)				Extremely important
	**Overall health, wellbeing and life**	1	2	3	4	5	6	7	8	9
128	Normality (feeling able to live a ‘normal’ life)	1	2	3	4	5	6	7	8	9
129	Feeling in control of health and wellbeing	1	2	3	4	5	6	7	8	9
130	Having a positive outlook on life and expectations for the future	1	2	3	4	5	6	7	8	9

SECTION 6 Additional Issues

Please write down any issues relating to obesity surgery not included above that you think are important to measure in research studies. We welcome all your views.

Thank you for your contribution to this round of the survey. We look forward to providing you with the results in the next round!

Contact details
Department of Social Medicine
FREEPOST (BS.4826)
Bristol
BS8 2YX

Katie.whale@bristol.ac.uk
